# Economic impact of biomarker-based aging interventions on healthcare costs and individual value

**DOI:** 10.3389/fgene.2026.1814599

**Published:** 2026-10-12

**Authors:** Federico Felizzi

**Affiliations:** enGene, Basel, Switzerland

**Keywords:** aging biomarkers, DunedinPACE, epigenetic clock, frailty, healthcare costs, longevity medicine, willingness to pay

## Abstract

We investigate the economic impact of controlling the pace of aging through biomarker monitoring and targeted interventions. Using the DunedinPACE epigenetic clock as a measure of biological aging rate, we model how different intervention scenarios affect frailty trajectories and their subsequent influence on healthcare costs, lifespan, and health quality. Our model demonstrates that controlling DunedinPACE from age 50 onward can reduce frailty prevalence, resulting in cumulative healthcare savings of up to CHF 131,608 per person over 40 years in our most optimistic scenario. From an individual perspective, the willingness to pay for such interventions reaches CHF 3.2 million in the most optimistic scenario when accounting for both extended lifespan and improved health quality. When the direct cost of epigenetic testing is included, the healthcare-system case is sensitive to test price and testing frequency, whereas the individual-value case remains strongly positive. These findings suggest substantial economic value in technologies that can monitor and modify biological aging rates, providing evidence for both healthcare systems and consumer-focused business models in longevity medicine.

## 1 Introduction

Population aging presents unprecedented socioeconomic challenges worldwide (Ruckstuhl et al., 2023), while simultaneously creating new opportunities for artificial intelligence-driven digital health solutions focused on the aging process. This study investigates potential business models to address challenges related to aging and longevity medicine in real-world settings. We explore whether a combination of treatments, monitoring programs, and lifestyle interventions can meaningfully influence clinical outcomes related to aging and reduce costs associated with caregiving and diminished work capacity during reduced healthspan periods.

When establishing endpoints for longevity interventions, we draw upon the comprehensive framework presented by Cummings and Kritchevsky (2022), which outlines various endpoints including total mortality, cause-specific mortality, and composite measures. We acknowledge the inherent challenges in establishing surrogacy between intermediate endpoints and outcomes of interest, particularly regarding statistical power and necessary follow-up duration.

Among potential endpoints, frailty-related measures such as the Frailty Index (Rockwood et al., 2005) show particular promise for our objectives because: (1) frailty demonstrates direct correlation with healthcare expenditures (Hoogendijk et al., 2019), and (2) numerous studies have established robust relationships between frailty, all-cause mortality, and major comorbidities including cancer, dementia, and cardiovascular disease.

A promising biomarker for measuring biological age is the DunedinPACE clock, which quantifies the pace of epigenetic change through DNA methylation patterns. Recent research demonstrates that DunedinPACE can predict frailty development and chronic disease onset more effectively than chronological age or alternative biomarkers (Mak et al., 2023). This clock captures several distinct influences on the rate of biological aging. A faster pace of aging has been observed in young adults with histories of early-life adversity, including childhood socioeconomic disadvantage and victimization (Belsky et al., 2020; 2022), and in children exposed to perinatal adversity (Barr et al., 2025). Twin studies indicate a substantial but age-dependent genetic contribution to the pace of aging (Hong et al., 2024). Prospectively, a faster DunedinPACE predicts incident chronic disease, disability, accelerated brain atrophy, and all-cause mortality across multiple cohorts (Belsky et al., 2022; Vetter et al., 2026; Bourassa et al., 2025; Whitman et al., 2025). We note, however, that DunedinPACE reflects only DNA-methylation signatures and does not capture other hallmarks of aging (e.g., telomere attrition and loss of proteostasis); it should therefore be interpreted as one informative axis of biological aging rather than a comprehensive summary.

In this manuscript, we propose a novel biomarker-based monitoring and intervention strategy for longevity medicine based on routine DunedinPACE assessments in clinical settings. We hypothesize that measuring individuals’ pace of aging enables identification of those at risk for accelerated aging, allowing for targeted preventive measures and personalized interventions to slow the aging process and improve health outcomes. We estimate the economic implications of this approach through a predictive model simulating different personalized recommendation scenarios designed to optimize epigenetic clock trajectories.

Our analysis establishes connections between DunedinPACE, frailty progression, and mortality outcomes (Figure 1) to estimate:

1. The impact of interventions (at varying efficacy levels) on total healthcare system costs
2. Individual willingness-to-pay at different ages based on anticipated improvements in healthspan and lifespan
3. Quality-adjusted life year (QALY) improvements resulting from changes in quality-of-life utility and lifespan extension

**FIGURE 1:**
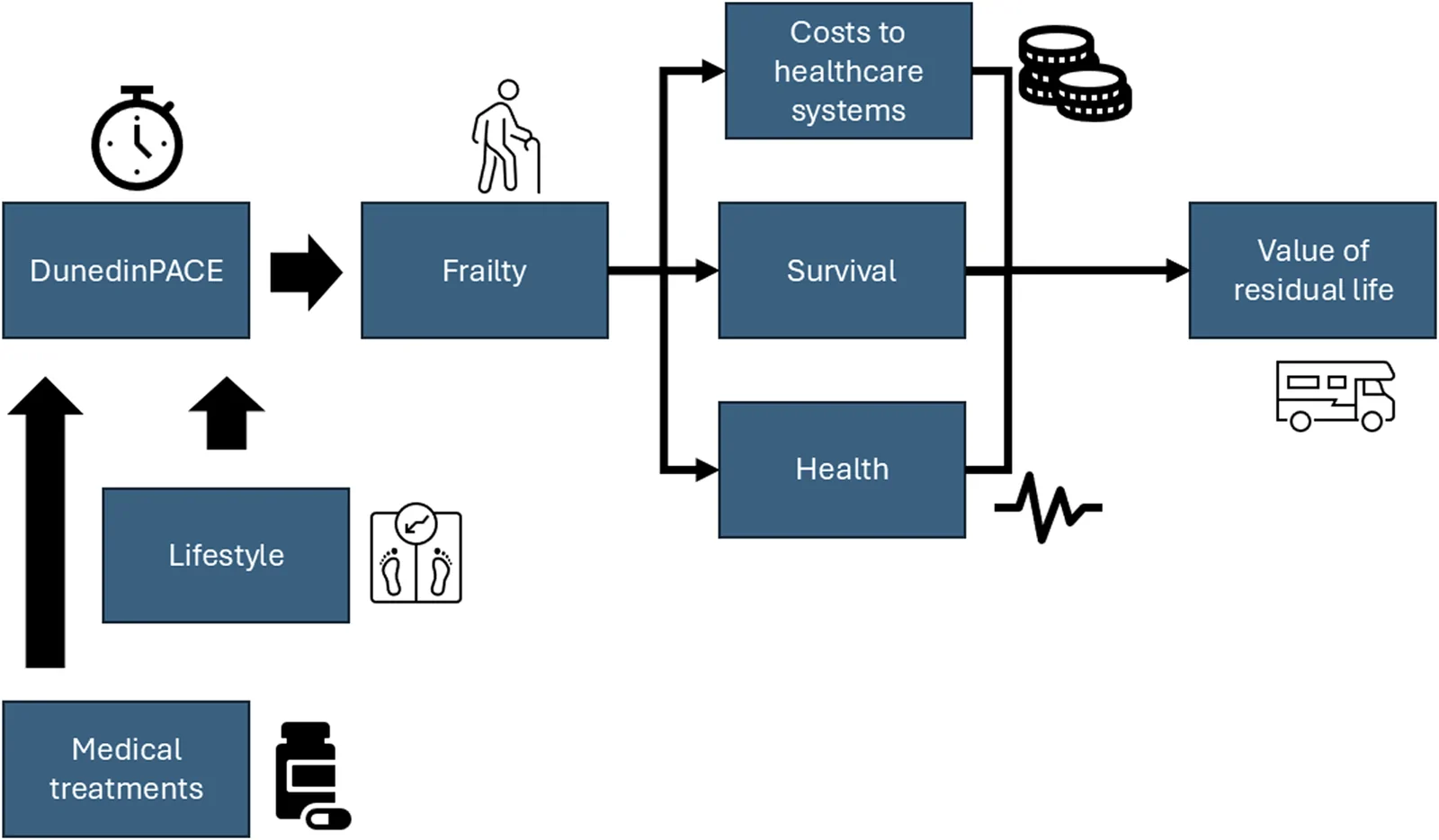
Conceptual overview of the modeling framework. Interventions that modify the pace of aging, measured by the DunedinPACE epigenetic clock, whether pharmacological or lifestyle-based, slow the age-related rise of the population Frailty Index. Lower frailty propagates into three linked outcomes: reduced direct healthcare-system costs, higher survival, and better health-related quality of life. Together, these raise the individual value of residual life, connecting a molecular biomarker to health-economic endpoints.

Through this approach, we aim to develop an economically viable business model that leverages digital technology and epigenetic monitoring to extend healthy lifespan through prevention and treatment of age-related conditions.

## 2 Methods

### 2.1 Review of the endpoints used in longevity trials

We began with a PubMed and Google Scholar review of the main trials used in longevity medicine to identify the key endpoints relevant for our analytic model and decision-making framework. We did not perform a systematic review, because the population, intervention, comparator, and outcomes (PICOS) criteria are very broad, so the number of hits would be too large to manage. Our target population includes all adults, with no age limit. The intervention of interest is any drug, monitoring approach (including digital health), or lifestyle change. The comparator is any alternative, which can mean either maintaining the current lifestyle or worsening it. The outcomes include endpoints such as death, the occurrence of specific diseases, or quality-of-life questionnaires. The large scope of the search terms led us to conduct a focused review, based on key studies illustrating endpoints previously used in trials targeting longevity. We relied on two reviews to help choose the trials and endpoints to consider. One article explains the pros and cons of longevity-relevant endpoints and provides recommendations on trial designs in longevity and their relevant endpoints. It also discusses potential surrogate endpoints, which may help address the limitations of traditional clinical endpoints that require long follow-up periods and large numbers of events to achieve sufficient statistical power (Cummings and Kritchevsky, 2022). A more recent study builds on this work and proposes new biomarker-related endpoints that can serve as surrogates for mortality and age-related functional decline. The authors present a list of ongoing or completed trials, with interventions ranging from pharmacological to lifestyle changes, and novel endpoints, such as those linked to DNA methylation (Moqri et al., 2023).

### 2.2 Frailty Index and its relationship to the prevalence of frailty

The Frailty Index is a comprehensive metric designed to assess the vulnerability of older adults by quantifying their health status across a spectrum of clinical deficits. Typically, this index encompasses a wide range of health deficits, often 30 to 40, including symptoms, diseases, disabilities, and laboratory abnormalities. Each deficit is evaluated, and the index is calculated by dividing the number of deficits present in an individual by the total number assessed, yielding a score between 0 and 1, following the standard construction procedure (Searle et al., 2008). A higher score indicates a higher degree of frailty.

The clinical definition of frailty is based on established cutoff scores. Individuals with a Frailty Index of 0.25 or higher are typically labeled “frail,” signifying a high risk of adverse health events, while an index between 0.10 and 0.24 usually places an individual in the “pre-frail” category. We emphasize that these cut-points are conventional operationalizations rather than sharp biological boundaries (Searle et al., 2008). We also note that the Frailty Index counts each deficit equally and does not weight deficits by clinical severity, so that conditions of very different prognostic weight each contribute a single count. Recent work proposing a severity-weighted, multi-system alternative found that it captured health heterogeneity that the Frailty Index misses and outperformed it in predicting disability and mortality (Salimi et al., 2025). This is a recognized limitation of the cumulative-deficit approach, bounded by the fact that the index was constructed so that its association with adverse outcomes is robust to the particular deficits chosen when a sufficient number are included (Mitnitski et al., 2001; Searle et al., 2008). We return to its implications for our economic outcomes in Section 4, Discussion.

The distribution of the Frailty Index across age groups enables the estimation of the proportion of subjects considered frail or pre-frail within each group. When frailty is operationalized as a Frailty Index above a fixed threshold, its prevalence at a given age follows from the underlying deficit-accumulation distribution by construction; several studies have characterized how that distribution shifts with age. Rockwood et al. characterized the distribution of the Frailty Index in humans and mice as a function of age and related incremental Frailty Index scores to an all-cause-mortality hazard ratio (Rockwood et al., 2017). Rockwood and Howlett later showed that the Frailty Index distribution follows a gamma distribution at younger ages, with a long right tail, and becomes more symmetric with age (Rockwood and Howlett, 2019). These observations were confirmed by a longitudinal study in Korea, which characterized the evolution of Frailty Index distributions with age and reported a median Frailty Index of approximately 0.5 among females aged 95–98 (Baek and Min, 2022). To determine the proportion of frail people at a given age, we compute the proportion of subjects above the 0.25 Frailty Index threshold, in accordance with the definition. We perform a normal approximation of the gamma distribution and calculate the proportion of frail subjects at each age as

$$\pi_{t}^{\mathrm{frail}}=1-\Phi \left(\frac{0.25-{FI}_{t}}{\sigma_{t}^{FI}}\right),$$ (1)

with $\pi_{t}^{\mathrm{frail}}$ indicating the proportion of frail subjects at age t , ${FI}_{t}$ as the Frailty Index at age t , $\sigma_{t}^{FI}$ as the standard deviation of the Frailty Index at age t , and Φ(⋅) as the cumulative distribution function of a standard normal distribution.

### 2.3 Model development

In this section, we explain the data sources and methods used to meet the goals of the manuscript. One main challenge of using frailty as an endpoint is that it occurs relatively late in life, with trials such as DO-HEALTH and ASPREE enrolling subjects older than 65–70 years (McNeil et al., 2018; Bischoff-Ferrari et al., 2020). Finding studies targeting individuals younger than 65 is therefore crucial. Several studies, including the CALERIE trial, reported the effects of dietary interventions on younger subjects (Waziry et al., 2023). The work by Mak et al. (2023) modeled the evolution of the *Frailty Index* as a function of the evolution of five epigenetic clocks, including clocks that assess the current estimate of biological age (PCHorvathAge, PCHannumAge, PCPhenoAge, PCGrimAge) and the DunedinPACE clock, which assesses the pace of aging. The study focuses on subjects older than 50 years, enabling evidence generation for more than one additional decade than earlier studies (McNeil et al., 2018). From a modeling standpoint, the study used a Dual-Change-Score Model (Wiedemann et al., 2022) to model the dependencies between the Frailty Index and the epigenetic clocks. Importantly, the study demonstrated how changes in the proportion of frail subjects in a population are influenced by the current proportion of frail individuals and by the value of a specific epigenetic clock.

The study concluded that there is no statistically significant relationship between the Frailty Index and any of the clocks measuring the estimate of biological age, but that there is a dependency between frailty and DunedinPACE, with a one-directional coupling effect DunedinPACE → frailty (reported in Table 1).

**TABLE 1:** Parameters employed in the model with corresponding values.

Parameter	Description	Value
α	​	1
$FI_{50}$	Frailty index at 50 years	6.55 (%)
$FI_{50}^{\mathrm{gender}}$	Effect of gender (female vs. male) on $FI_{50}$	0.60^*^
$FI_{S}$	Slope of frailty	−12.31
$FI_{S}^{\mathrm{gender}}$	Effect of gender (female vs. male) on $FI_{S}$	0.29^*^
$\beta_{FI}$	Coefficient on FI	0.06
$\gamma_{\mathrm{Clock\to FI}}$	Coupling coefficient between clock (DunedinPACE) and FI	1.19
$Clock_{50}$	10× value of DunedinPACE at age 50	10.04
$\beta_{\mathrm{Clock}}$	​	0.06
$HR_{\frac{frail}{robust}}$	Hazard ratio in all-cause mortality between frail and robust people	2.40

* Reported in Mak et al. (2023) but not used in Equations 2–5 of the present model.

The model structure identified by Mak et al. (2023) is described by Equation 2:

$${\Delta FI}_{t}=\alpha \cdot {FI}_{S}+\beta_{FI}\cdot {FI}_{t-1}+\gamma_{\mathrm{Clock\to FI}}\cdot {Clock}_{t-1},$$ (2)

where a 2-year cycle is assumed (the time gap between consecutive cycles t−1 and t is 2 years), and the parameters are outlined in Table 1. The change in DunedinPACE over time is modeled as a linear function of age:

$${Clock}_{t}=\beta_{\mathrm{Clock}}+{Clock}_{t-1}.$$ (3)

We extend the quantitative model by Mak et al. (2023) to determine how changes in the DunedinPACE profile affect changes in the Frailty Index over a 40-year period, between 50 years and 90 years of age.

We construct three types of evolution of the DunedinPACE clock, depending on assumptions about the evolving evidence base:

1. Interventions such as caloric restriction (as in the CALERIE study) reduce DunedinPACE by 0.01 relative to the expected age-dependent evolution, a simple downward shift along the y-axis (Figure 2) [CR0].
2. In addition to a y-axis shift, interventions reduce the slope of the natural evolution of DunedinPACE, lowering the year-on-year growth of the pace of aging [CR1].
3. A spectrum of interventions sets the pace of aging exactly at 1.0 (the natural pace) or slightly below [CR2].

**FIGURE 2:**
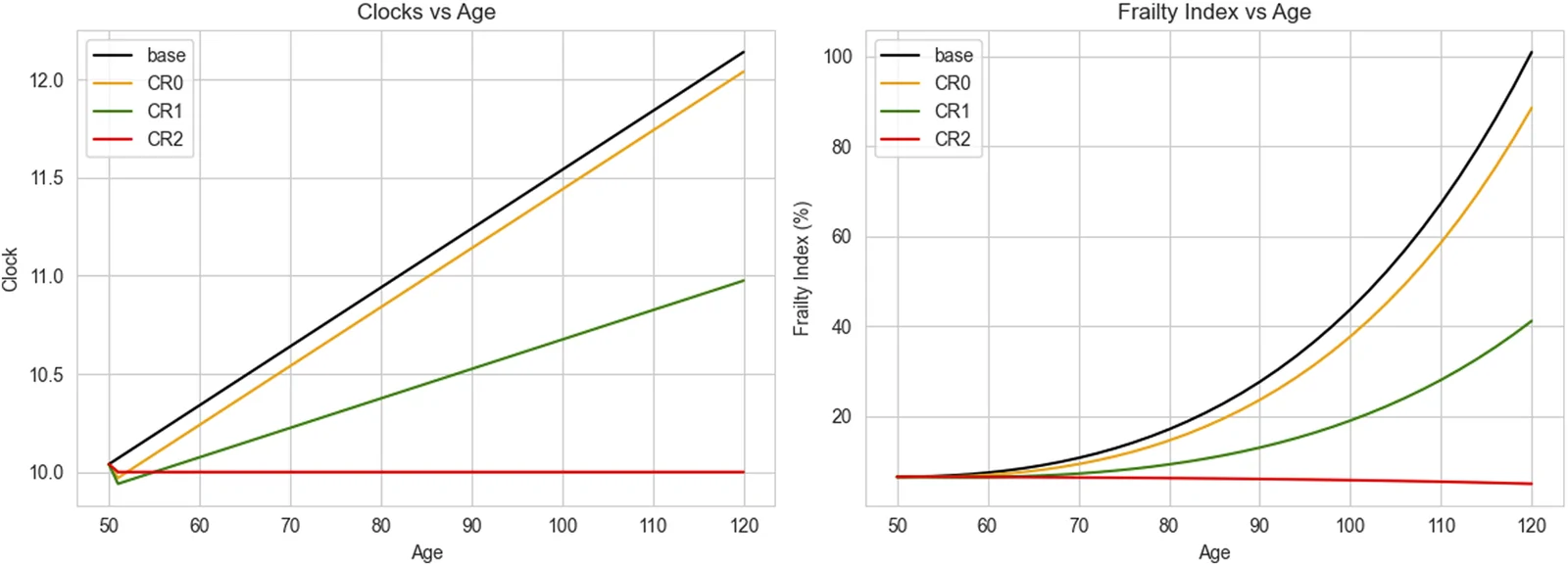
Effect of the three intervention scenarios on the pace of aging (left panel) and the resulting Frailty Index (right panel) from age 50. Baseline (black) shows the natural increase in DunedinPACE and the accelerating Frailty Index. CR0 (orange) applies a constant downward shift of 0.01 to the clock, as implied by the CALERIE caloric-restriction trial; CR1 (green) additionally reduces the slope of the clock trajectory; and CR2 (red) holds the pace of aging at 1.0. Each successively stronger control of the clock (left) translates into a lower, flatter Frailty Index trajectory (right).

Interventions are modeled as sustained across the 50–90 horizon (rather than one-off exposures); their direct costs, together with the cost of epigenetic measurement, are incorporated on the cost side of the analysis as described in Section 2.8. The three scenarios are linked to the evolution of the *Frailty Index* as depicted in Figure 2. As outlined in Section 2.2, the link between the Frailty Index and the prevalence of frailty is governed by Equation 1. After calibrating the standard deviation $\sigma_{t}^{FI}$ to match the proportion-of-frailty curves observed in an international study presenting Dutch data (Hoogendijk et al., 2019), we obtain the curves in Figure 3.

**FIGURE 3:**
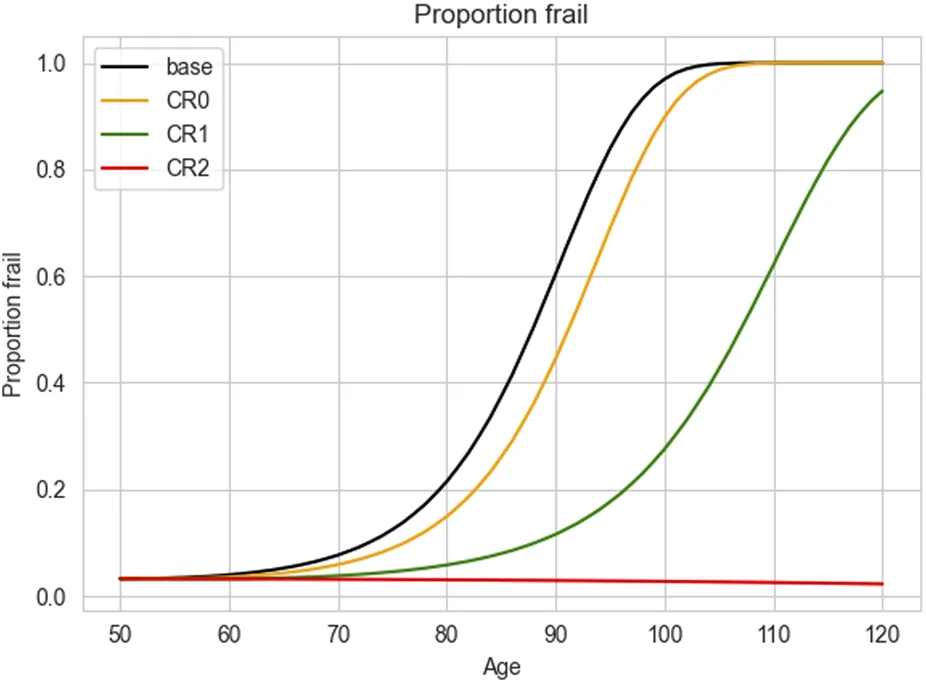
Proportion of the population classified as frail (Frailty Index >0.25 ) as a function of age, by scenario, obtained by integrating the age-specific Frailty Index distribution above the 0.25 threshold (Equation 1). Relative to baseline (black), each intervention lowers the frail fraction at every age, with the largest reduction under CR2 (red); the gap between baseline and the intervention scenarios widens in later life, when frailty is most prevalent.

### 2.4 Mortality and survival modeling

Survival curves were derived using a step-wise time-dependent hazard model that accounts for the changing frailty status of individuals over time. The baseline mortality hazard was calibrated to Swiss life tables, while the frailty-dependent component used the hazard ratio of 2.40 between frail and robust individuals established in previous studies (Rockwood et al., 2017). At each age, the probability of survival was calculated as

$$S\left(t+1|t\right)=S\left(t\right)\times \left(1-h\left(t\right)\right),$$ (4)

where h(t) is the age- and frailty-dependent hazard rate at age t , modeled as

$$h\left(t\right)=h_{\mathrm{baseline}}\left(t\right)\times \left(1+\left({\text{HR}}_{\mathrm{frail∕robust}}-1\right)\times \pi_{t}^{\mathrm{frail}}\right).$$ (5)

### 2.5 Healthcare cost estimation

Healthcare cost data were primarily derived from a Dutch study examining annual healthcare expenditures for frail versus non-frail individuals (Hoogendijk et al., 2019). The study reported annual costs of EUR 30,792 for frail individuals and EUR 15,611 for non-frail individuals in 2015, which we adjusted to 2024 Swiss Francs accounting for inflation and purchasing power parity, yielding CHF 52,168 and CHF 26,448, respectively (Table 2).

**TABLE 2:** Healthcare costs of frail vs. non-frail individuals across countries, with adjustments to 2024 Swiss costs (CHF) accounting for inflation and purchasing power.

Country	Year	Annual cost		2024 CHF adjusted	
​	​	Frail	Non-frail	Frail	Non-frail
Netherlands	2015	EUR 30,792	EUR 15,611	52,168	26,448
Australia	2016	AUD 28,906	AUD 19,905	​	​
United States of America	2011	USD 76,363	USD 27,731	​	​

While the Dutch study reports a direct impact on total annual healthcare costs, which translates readily into potential savings for decision-makers, the US and Australian studies focus on post-operative costs for selected categories and are difficult to translate into direct healthcare-system savings. A Swiss study calculated the proportion of people admitted to hospital by frailty status, showing that the 10-year incidence of hospital admission is approximately six-fold higher in frail or pre-frail people than in robust individuals (Luini and Calciolari, 2023). However, because that study focuses on a hospitalized population, we consider its findings not directly applicable to our context.

### 2.6 Value to individuals

We outline the economic framework to value improvements in lifespan and healthspan, following Murphy and Topel (2006), Scott (2023), and Scott et al. (2023). Lifetime utility for an individual of age a is defined as Equation 6:

$$\mathrm{utility}\left(a\right)=\int_{a}^{\infty }H\left(t\right)u\left(c\left(t\right),l\left(t\right)\right)\tilde{S}\left(t,a\right)e^{-\rho \left(t-a\right)}dt,$$ (6)

with H(t) as the intangible value of health affecting quality of life without affecting mortality; u(c(t),l(t)) as the utility to an individual, depending on consumption c(t) and non-market/leisure time l(t) ; $\tilde{S}\left(t,a\right)$ as the probability of survival from age a to t ; and ρ as the discount rate. Under a perfect annuity market, the expected discounted value of future consumption equals expected wealth, as demonstrated by Equation 7 (Murphy and Topel, 2006):

$$A\left(a\right)+\int_{a}^{\infty }\left[y\left(t\right)-c\left(t\right)\right]\tilde{S}\left(t,a\right)e^{-r\left(t-a\right)}dt=0,$$ (7)

with A(a) representing the initial assets at age a , r representing the interest rate, and y(t) representing the life-contingent income defined in Equation 8,

$$y\left(t\right)=w\left(t\right)\left[1-l\left(t\right)\right]+b\left(t\right),$$ (8)

where w(t) is the wage per period, l(t) is the proportion of leisure time, and b(t) is the non-wage income (e.g., a pension). The value of residual life at age a is

$$V\left(a\right)=\int_{a}^{\infty }v\left(t\right)\tilde{S}\left(t,a\right)e^{-r\left(t-a\right)}dt,$$ (9)

independent of the rate of time preference ρ , where v(t) is the value of a life year at age t≥a , itself a function of the wage, working hours, leisure time, and the marginal utility of consumption (Murphy and Topel, 2006; Scott et al., 2021). The willingness to pay for a technological advancement ξ (e.g., information from epigenetic clocks measuring the pace of aging, inducing lifestyle changes, or pharmaceuticals targeting the aging process) is

$${WTP}_{\xi }\left(a\right)=\underset{\text{lifespan}}{\underbrace{\int_{a}^{\infty }v\left(t\right)\Delta_{\xi }S^{*}\left(t,a\right)dt}}+\underset{\text{healthspan}}{\underbrace{\int_{a}^{\infty }\frac{\Delta_{\xi }H\left(t\right)}{H\left(t\right)}\frac{u\left(c\left(t\right),l\left(t\right)\right)}{u_{c}\left(c\left(t\right),l\left(t\right)\right)}dt}},$$ (10)

where $S^{*}$ is discounted survival, $\Delta_{\xi }S^{*}$ and $\Delta_{\xi }H$ denote the changes in survival and health induced by ξ , and $u_{c}$ is the marginal utility of consumption. The two independent terms represent the contributions to willingness to pay from changes in lifespan and healthspan, respectively.

### 2.7 Linking frailty to health

Scott et al. (2021) modeled slower aging as a joint improvement in mortality and frailty. We model health as a linear function of the Frailty Index, consistent with prior studies that describe the inverse relationship between the Frailty Index and health-related quality-of-life utility (Nathiya et al., 2023). We simplify this to H(t)=1−FI(t) . We measure model outcomes in terms of the number and proportion of individuals who are frail, have chronic diseases, or die at each age.

### 2.8 Cost of epigenetic measurement and discounting

Because health-economic evaluations should be reported net of monitoring and intervention costs, we incorporate the direct cost of DunedinPACE testing. We assume a price of CHF 600 per assessment, administered twice per year (CHF 1,200 per year), incurred only while the individual is alive; the cumulative testing cost therefore scales with the survival curve. This monitoring-and-intervention strategy is assumed to produce the moderate biological response of scenario CR1. Unless otherwise stated, reported healthcare savings and individual value are given both gross and net of this cost. We report both undiscounted results and results under standard health-economic discounting, applying $1/{\left(1+r\right)}^{\left(\text{age}-50\right)}$ with r=3.5% per year for cost cash-flows and r=1.5% per year for outcome flows (QALYs and survival/health value).

## 3 Results

Unless otherwise noted, all monetary figures are per individual and undiscounted, and intervention scenarios are compared against the baseline (natural-aging) projection.

### 3.1 Trajectories of the pace of aging and the Frailty Index

Figure 2 shows the modeled evolution of the DunedinPACE clock (left panel) and the Frailty Index (right panel) from age 50 to 90. Under baseline aging, DunedinPACE increases with age, and the Frailty Index accelerates in later life. The CR0 scenario shifts the clock trajectory downward by a constant offset; CR1 additionally reduces its slope; and CR2 holds the pace of aging at 1.0, producing the most favorable Frailty Index trajectory. The corresponding age-specific distributions of the Frailty Index (Figure 4) shift rightward and widen with age, with an increasing fraction of the distribution crossing the 0.25 frailty threshold.

**FIGURE 4:**
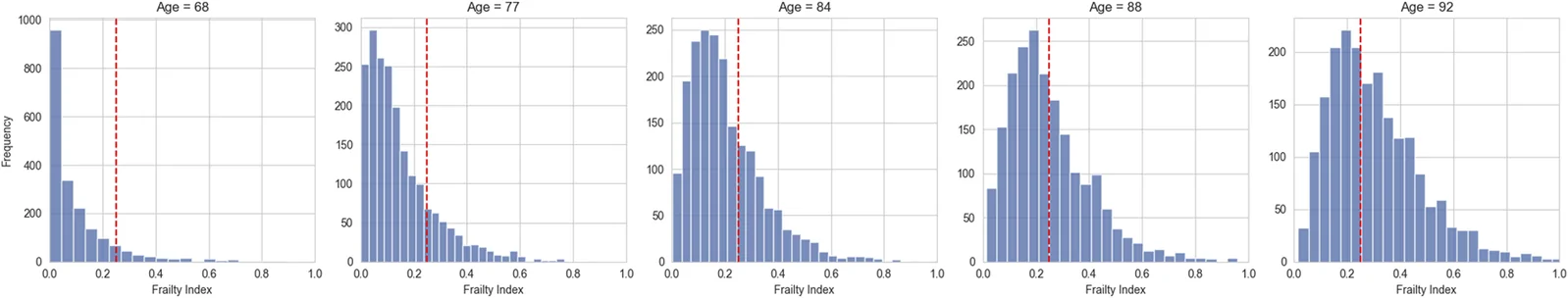
Age-dependent distribution of the Frailty Index under the baseline scenario. Each panel shows the simulated population distribution at a successively older age (left to right), modeled as a gamma distribution whose mean is taken from the data and whose variance widens with age. The vertical dashed red line marks the 0.25 threshold above which an individual is classified as frail. As age increases, the entire distribution shifts rightward and broadens, so that a progressively larger share of the population crosses the threshold. This mechanism explains how frailty prevalence increases with age in the model.

### 3.2 Prevalence of frailty

Applying Equation 1 to the scenario-specific Frailty Index trajectories yields the proportion of frail individuals as a function of age (Figure 3). Relative to baseline, all three intervention scenarios decrease the proportion of frail subjects at every age, with the largest reductions under CR2, where the pace of aging is held at its natural rate.

### 3.3 Survival and life expectancy

The frailty-dependent hazard model (Equations 4, 5) produces the survival curves in Figure 5. Reduced frailty prevalence translates into improved survival and gains in life expectancy relative to baseline of 0.77, 2.89, and 3.58 years for CR0, CR1, and CR2, respectively (Table 3).

**FIGURE 5:**
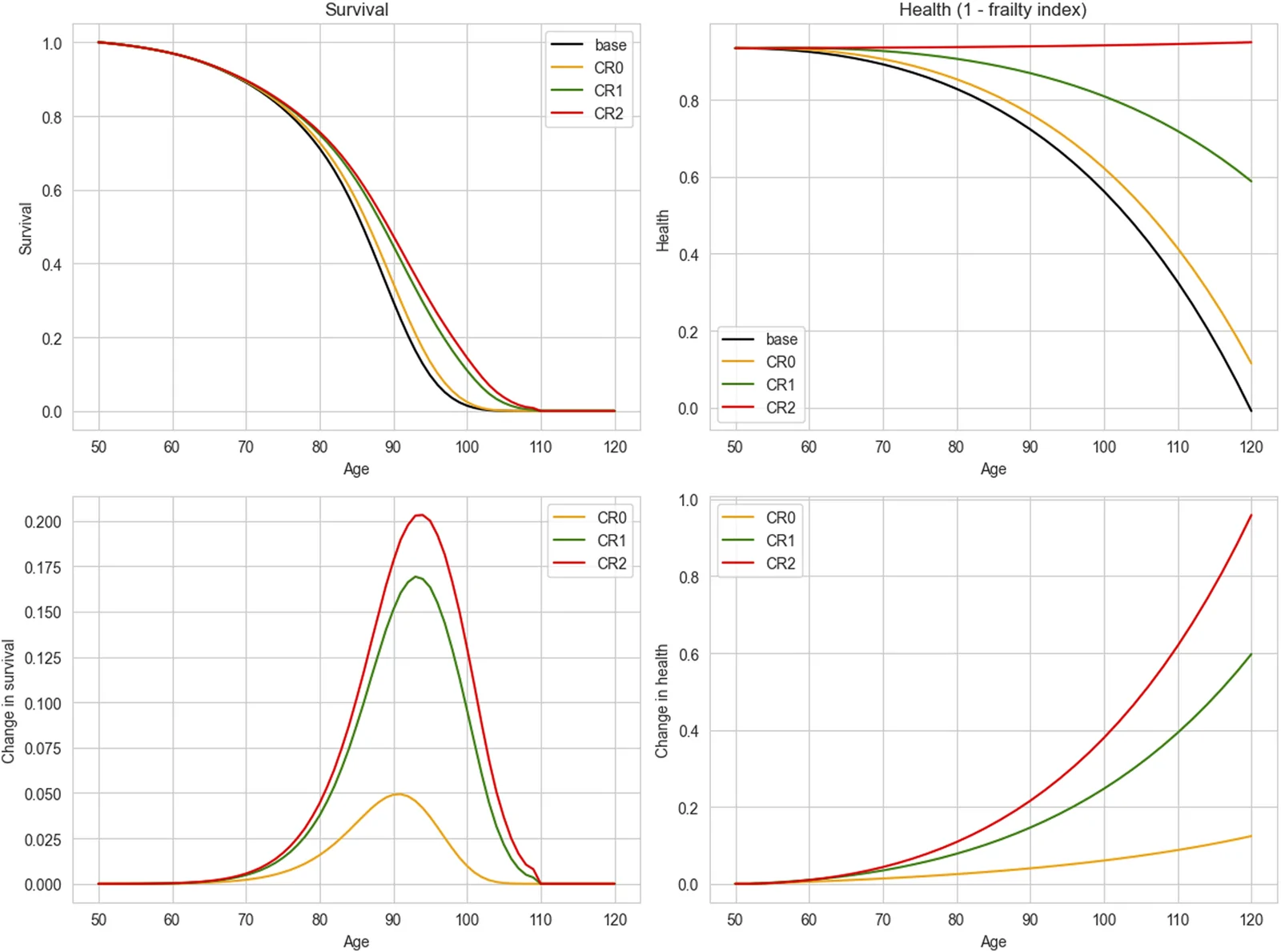
Survival curves from age 50 under each scenario, derived from a frailty-dependent hazard model (Equations 4, 5) with a frail-to-robust mortality hazard ratio of 2.40 calibrated to Swiss life tables. Lowering frailty reduces the age-specific hazard and shifts survival rightward, corresponding to life-expectancy gains of 0.77 (CR0), 2.89 (CR1), and 3.58 (CR2) years relative to baseline.

**TABLE 3:** Headline outcomes by intervention scenario (gross of testing cost). Healthcare savings are cumulative to age 90 under cost-weighting Scenario 1; WTP_surv_ is the survival component of willingness-to-pay; Δ QALY is the undiscounted QALY gain relative to baseline.

Scenario	LE gain (yr)	Healthcare savings (CHF)	WTP_surv_ (CHF)	Δ QALY
CR0	0.77	43,676	274,675	1.07
CR1	2.89	112,427	986,781	3.90
CR2	3.58	131,608	1,216,186	5.32

### 3.4 Healthcare-system cost savings

Annual healthcare costs by scenario and cumulative savings are shown in Figure 6. Over the 40-year horizon (ages 50–90), cumulative savings reach CHF 43,676 (CR0), CHF 112,427 (CR1), and CHF 131,608 (CR2) per individual (Table 3). Savings accrue progressively and are largest in the ninth decade of life, reflecting the concentration of frailty-attributable costs in late life under a sustained intervention rather than a delayed effect of a brief exposure.

**FIGURE 6:**
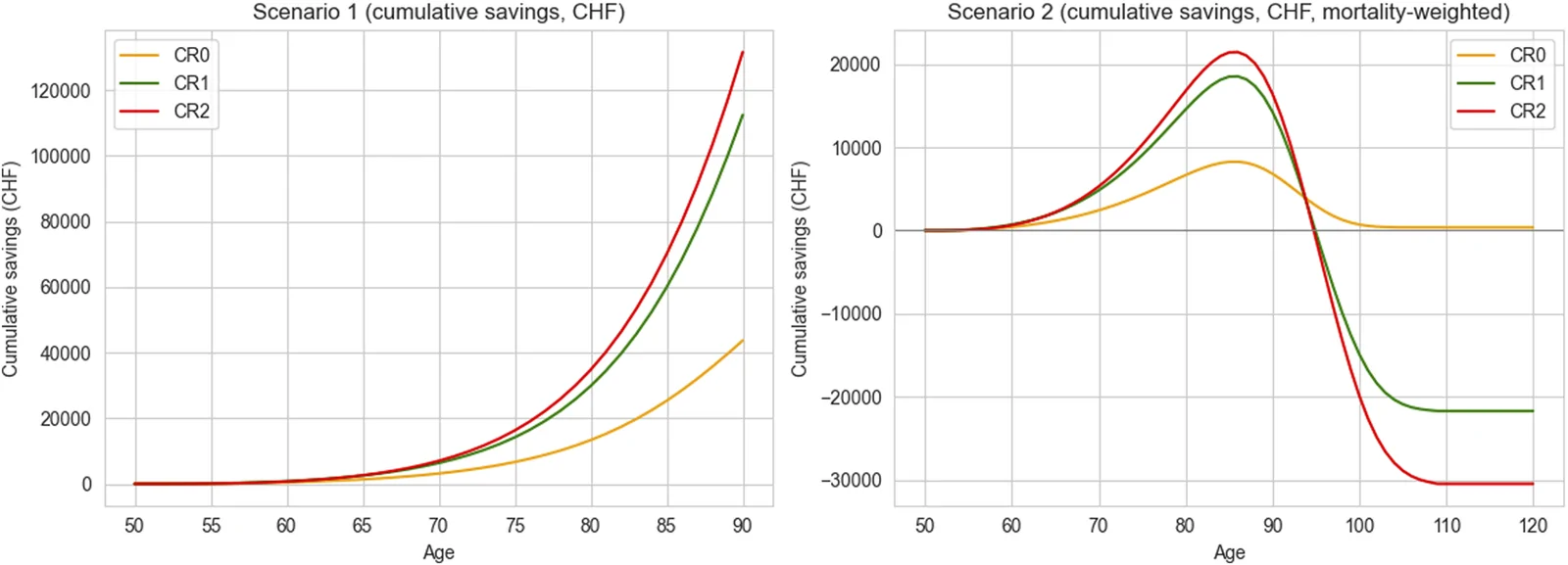
Cumulative per-person healthcare savings relative to baseline, by scenario. Left (Scenario 1): savings from the reduced frail fraction, valued at the difference in annual cost between a frail (CHF 52,168) and a robust (CHF 26,448) individual, cumulated to age 90. Right (Scenario 2): the same savings weighted by survival, so that healthcare costs incurred during the additional life-years gained under each intervention offset earlier savings; cumulative savings peak in the late 80s and turn negative for CR1 and CR2 thereafter.

### 3.5 Quality-adjusted life years

Accounting for both extended survival and improved health quality (H(t)=1−FI(t)) , the intervention scenarios yield 1.07, 3.90, and 5.32 additional QALYs for CR0, CR1, and CR2, respectively (Table 3). Evaluated at conventional cost-effectiveness thresholds of CHF 100,000–300,000 per QALY, the CR2 gain corresponds to a value of CHF 531,898–1,595,695 per individual.

### 3.6 Individual value and willingness-to-pay

Applying the value-of-life framework (Equation 10) with the weighting functions shown in Figure 7, individual willingness-to-pay decomposes into a survival component and a health-quality component (Figure 8; Table 4). Under CR2, the survival component is CHF 1,216,186, and the health-quality component is CHF 2,020,009, for a total individual value of CHF 3,236,195. The corresponding totals are CHF 705,662 (CR0) and CHF 2,484,305 (CR1).

**FIGURE 7:**
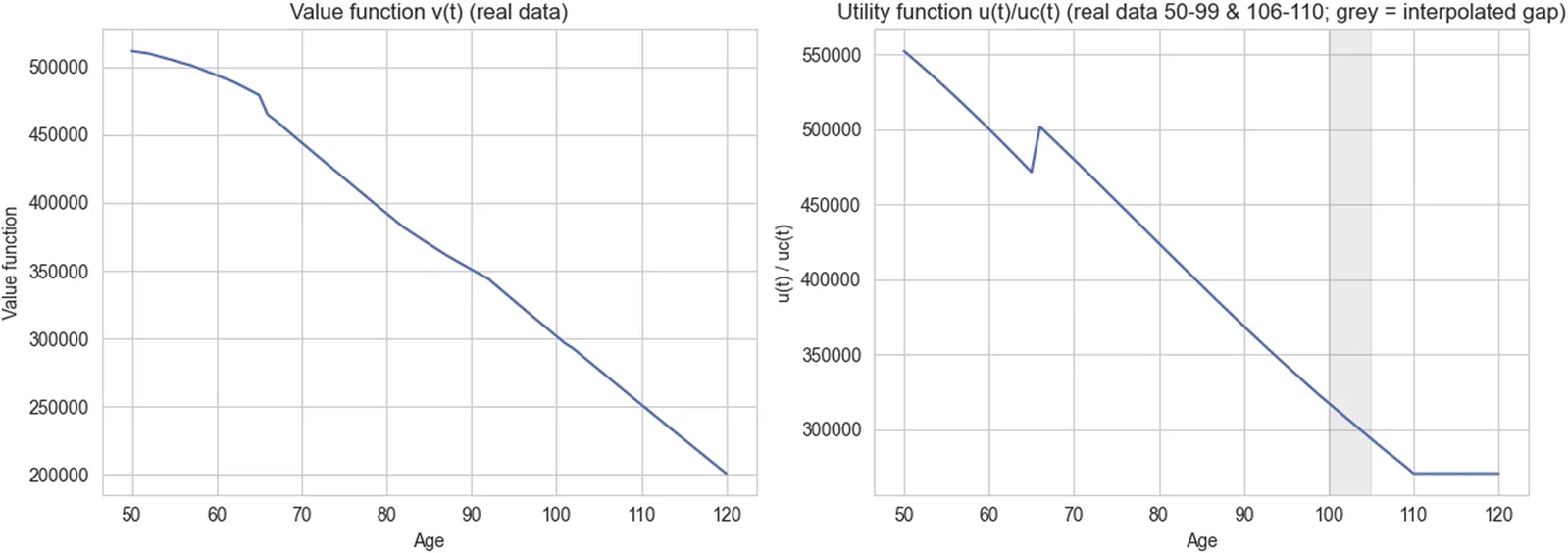
Two economic weighting functions that convert modeled changes in survival and health into monetary value (Equation 10). Left panel: the value of a life year, v(t) , which weights changes in *survival* to yield the lifespan component of willingness-to-pay. Right panel: the ratio of utility to the marginal utility of consumption, $u\left(t\right)/u_{c}\left(t\right)$ , which weights proportional changes in *health* quality to yield the healthspan component. Both profiles are drawn from real data; the shaded band marks the age range over which $u\left(t\right)/u_{c}\left(t\right)$ is interpolated.

**FIGURE 8:**
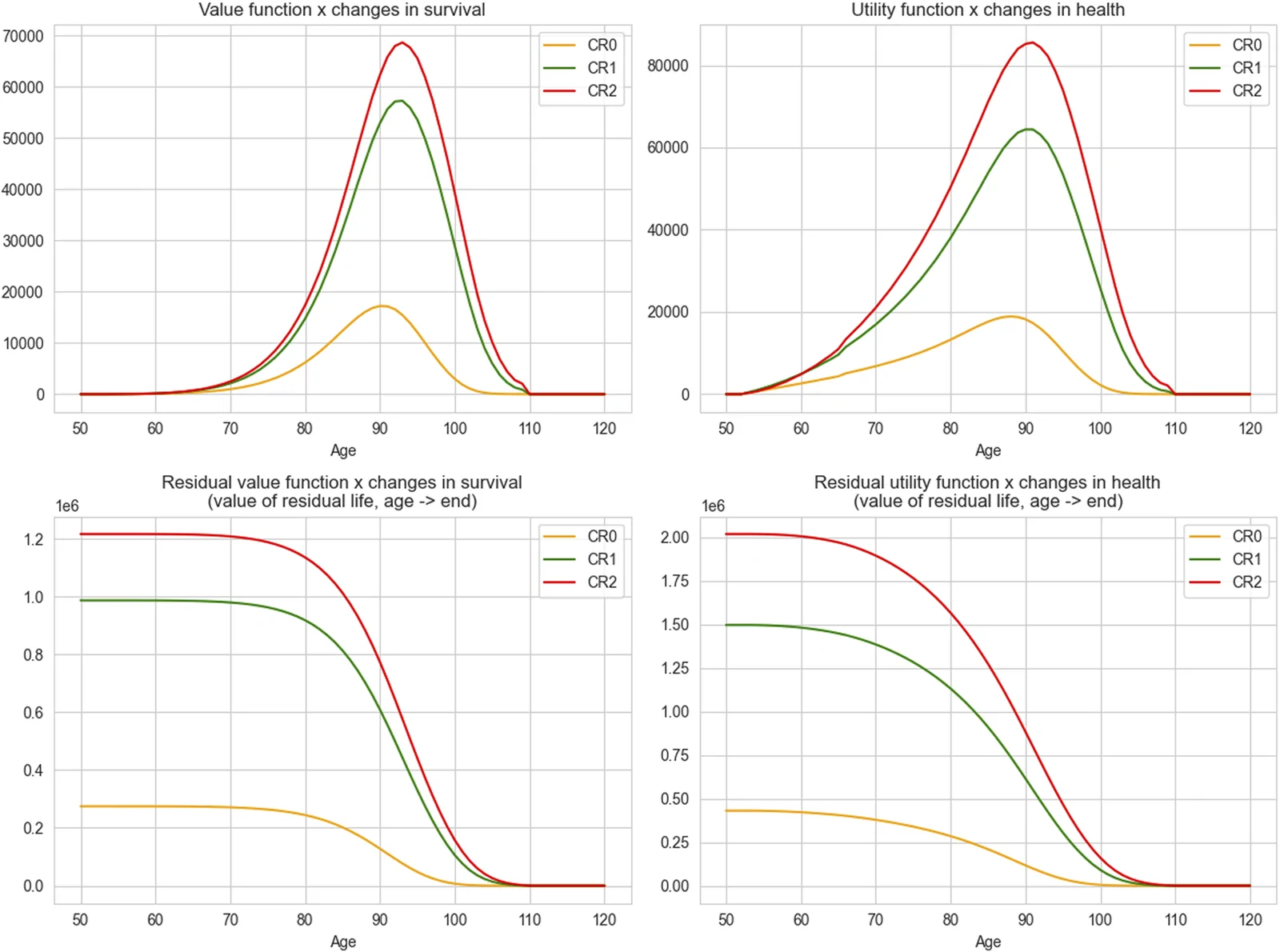
Contribution of survival and health-quality gains to individual willingness-to-pay, by scenario (Equation 10). Top row: the per-age value contributions: $v\left(t\right)\;\Delta_{\xi }S^{*}\left(t,a\right)$ for the lifespan (survival) component and $\frac{\Delta_{\xi }H\left(t\right)}{H\left(t\right)}\frac{u\left(c\left(t\right),l\left(t\right)\right)}{u_{c}\left(c\left(t\right),l\left(t\right)\right)}$ for the healthspan (health) component. Bottom row: the residual, reverse-cumulative value remaining from each age onward, that is, the value of residual life V(a) integrated from age a to the end of life (Equation 9). Integrated from age 50, the two components give the survival and health values whose scenario totals are reported in Table 4, for example, CHF 1,216,186 (survival) and CHF 2,020,009 (health) under CR2.

**TABLE 4:** Individual value of intervention (undiscounted), decomposed into the value of additional survival and the value of improved health quality.

Scenario	Survival value (CHF)	Health value (CHF)	Total value (CHF)
CR0	274,675	430,987	705,662
CR1	986,781	1,497,524	2,484,305
CR2	1,216,186	2,020,009	3,236,195

### 3.7 Net of the cost of epigenetic measurement

Table 5 reports scenario CR1 net of the cost of DunedinPACE testing, both undiscounted and discounted. From a healthcare-system perspective, netting out the CHF 1,200/year testing cost leaves a positive but modest saving under one cost weighting (Scenario 1: CHF 63,227 undiscounted; CHF 10,078 discounted) and a negative result under the more conservative weighting (Scenario 2: CHF − 27,062 undiscounted; CHF − 16,911 discounted). From an individual perspective, net willingness-to-pay remains large (CHF 2,439,637 undiscounted; CHF 1,392,839 discounted), because a CHF 1,200/year outlay is small relative to the valuation of additional quality-adjusted survival. The divergence of weak healthcare-system economics from strong individual economics at current test prices is itself a central finding for business-model design.

**TABLE 5:** Monitoring-and-intervention strategy (biological effect = CR1) net of DunedinPACE testing cost (CHF 600/test, twice yearly).

Quantity	Undiscounted (CHF)	Discounted (CHF)
Healthcare savings, scenario 1: gross	112,427	36,904
Less cumulative testing cost	49,200	26,826
**Healthcare savings, scenario 1: net**	**63,227**	**10,078**
**Healthcare savings, scenario 2: net**	− **27,062**	− **16,911**
Individual WTP (survival + health): gross	2,484,305	1,426,634
Less lifetime testing cost	44,668	33,795
**Individual WTP: net**	**2,439,637**	**1,392,839**
Δ QALY (CR1)	3.90	2.27

Scenario 1 and Scenario 2 denote the two healthcare-cost weightings; healthcare figures are cumulative to age 90. Individual WTP combines the survival and health-value terms. The bold values correspond to the total net savings, e.g. the gross benefit from the Healthcare savings or WTP calculations minus the lifetime testing costs.

### 3.8 Value of epigenetic measurement beyond the Frailty Index alone

Independent cohort evidence indicates that the epigenetic pace of aging carries prognostic information beyond standard clinical measures of frailty. In the Berlin Aging Study II, DunedinPACE was the strongest of 14 consensus aging biomarkers for 7-year mortality, whereas the frailty phenotype was not a statistically significant predictor in adjusted models (Vetter et al., 2026). DunedinPACE also adds incremental prediction of morbidity, disability, and mortality over the GrimAge clock (Belsky et al., 2022) and predicts future chronic-disease incidence and healthcare costs in large cohorts (Bourassa et al., 2025). These comparisons use the Fried frailty phenotype rather than the deficit-accumulation Frailty Index employed here and therefore establish that the pace of aging is independently informative rather than a direct index-versus-clock equivalence. Within our own framework, the incremental value of adding epigenetic monitoring to a Frailty Index-only strategy is the difference between the net figures in Table 5 and the corresponding Frailty Index-only baseline: positive but price-sensitive for the healthcare system, and strongly positive for the individual.

## 4 Discussion

In this section, we discuss the implications of our model and forecast results, address methodological strengths and limitations, outline implementation challenges, and propose directions for future research.

### 4.1 Summary of key findings

We have developed a novel biomarker-based monitoring and intervention strategy for longevity medicine based on routine DunedinPACE assessments in clinical settings. Our economic modeling demonstrates that DunedinPACE-based intervention scenarios yield benefits relative to baseline across three domains.

Healthcare systems could realize cost savings through delayed frailty onset and progression, with cumulative savings reaching CHF 131,608 per individual under the CR2 scenario over the 40-year cohort model (Table 3). These savings accrue progressively and are largest in the ninth decade of life, reflecting the concentration of frailty-attributable costs in late life under a sustained intervention. When the direct cost of DunedinPACE testing is netted out, however, the healthcare-system case becomes sensitive to test price and testing frequency, turning marginal to negative under conservative cost assumptions (Table 5).

Individual value assessments revealed considerable willingness-to-pay for interventions affecting the pace of aging, comprising CHF 1.22 million for survival improvements and CHF 2.02 million for health-related quality-of-life enhancements under the CR2 scenario, for a total individual value of approximately CHF 3.24 million (Table 4). This value remains strongly positive after netting out testing costs, suggesting robust economic incentives for personal investment in aging-moderation strategies.

Our QALY analysis showed meaningful gains across intervention scenarios (1.07–5.32 additional QALYs), translating into substantial value across accepted willingness-to-pay thresholds (CHF 100,000–300,000 per QALY).

The main implication is that epigenetic pace-of-aging assessments could provide a valuable framework for identifying accelerated aging trajectories and implementing targeted interventions, with an economic case that is strong at the individual level and, at the healthcare-system level, contingent on the cost of measurement.

### 4.2 Methodological strengths

Our approach leverages the DunedinPACE clock, a biomarker capturing the rate of epigenetic change with demonstrated predictive capacity for multiple age-related outcomes. It provides a dynamic assessment of the rate of biological aging that has been associated with early-life exposures and that prospectively predicts morbidity, functional decline, and mortality at the population level (Belsky et al., 2022), while capturing only DNA-methylation signatures rather than the full range of aging hallmarks.

Incorporating the Frailty Index as our primary clinical outcome allows translation between molecular-level epigenetic changes and clinically relevant functional status, providing a coherent pathway from biological processes to healthcare-utilization patterns. By linking these elements to economic metrics, such as healthcare costs, willingness-to-pay, and QALYs, we establish a comprehensive framework for evaluating aging-focused interventions. Furthermore, our analysis begins at age 50, approximately 15 years earlier than the inclusion criteria for most aging-targeted trials, potentially expanding the intervention window during which aging-modification strategies could yield meaningful benefits.

### 4.3 Limitations and constraints

Several limitations must be considered. First, our model relies on connecting results from disparate cohort studies. The dual-change-score model of Mak et al. (2023) linking DunedinPACE to Frailty Index progression was developed in a cohort distinct from the CALERIE trial that demonstrated caloric-restriction effects on DunedinPACE (Waziry et al., 2023). This cross-cohort extrapolation introduces uncertainty regarding generalizability. More broadly, our model combines parameters from heterogeneous sources, such as hazard ratios from Canadian deficit-accumulation cohorts and healthcare-cost data from a Dutch population. This introduces potential bias due to population heterogeneity in frailty distributions, hazard ratios, and healthcare costs across countries, and the absolute figures should be read as order-of-magnitude estimates rather than country-specific predictions.

Second, our intervention scenarios focus on caloric restriction as the primary mechanism for modifying DunedinPACE trajectories. While evidence supports this connection, it represents only one of many potential intervention pathways, and the CALERIE trial required sustained, closely supervised restriction that is not readily attained through routine lifestyle advice. Our model does not account for other lifestyle, pharmaceutical, or combination approaches that might influence the pace of aging through different mechanisms.

Third, our analysis uses frailty as the primary clinical outcome without explicitly modeling specific disease trajectories and therefore does not capture the heterogeneity of age-related pathologies across organ systems. A related limitation is intrinsic to the Frailty Index itself: computed as the proportion of deficits present, it assigns equal weight to every deficit irrespective of clinical severity, so that conditions of markedly different prognostic import each contribute a single count. Salimi et al. (2025) made this limitation explicit, showing that a severity-weighted, multi-system “Body Clock” captures health heterogeneity the Frailty Index misses. For example, individuals with identical Frailty Index values can have substantially different Body Clock values. The Body Clock supersedes the Frailty Index in predicting physical performance, disability, geriatric syndrome, and mortality (Salimi et al., 2025). This matters in our setting because healthcare costs and quality-of-life utility are driven by the severity and system distribution of deficits, not merely their count, so the linear mapping H(t)=1−FI(t) we adopt is a simplification. The limitation is partly offset by the robustness of the deficit-accumulation index to the particular deficits chosen when a sufficient number are included (Mitnitski et al., 2001; Searle et al., 2008), but it remains an important caveat when frailty is used as an aggregate economic outcome.

Fourth, our economic model necessarily simplifies the healthcare landscape, focusing on direct frailty-related costs while potentially omitting indirect expenditures. The analysis now incorporates the direct cost of epigenetic measurement (Section 2.8), but the healthcare-system conclusions are sensitive to the assumed test price (CHF 600) and testing frequency; a higher price such as the approximately USD 850 currently charged by some providers would further erode healthcare-system savings.

Finally, our model assumes a uniform response to intervention across individuals. In reality, response is heterogeneous: the baseline pace of aging is partly heritable (Hong et al., 2024), and individuals differ in their sensitivity to a given intervention and in adherence over time. Incorporating inter-individual variability, genetic modifiers of frailty trajectories, and explicit non-responder strata would yield a distribution of outcomes rather than a point estimate; the present results should therefore be read as cohort-average projections.

### 4.4 Future research directions

Expanded disease modeling: Future studies should extend beyond frailty to model how DunedinPACE and aging interventions affect specific age-related conditions, particularly neurological, cardiovascular, and musculoskeletal disease.

Additional value metrics: Beyond QALYs, incorporating disability-adjusted life years (DALYs) or quality-adjusted survival would provide a more complete assessment of value, especially given that extended lifespan can raise late-life healthcare costs.

Intervention diversity: Research should explore intervention modalities beyond caloric restriction, including exercise, sleep, stress reduction, pharmacological agents (e.g., metformin, rapamycin), and combinations, and evaluate their relative and synergistic effects on aging biomarkers.

Cohort validation: Validation studies should assess the DunedinPACE–frailty relationship across diverse populations to establish generalizability and identify subgroup variation.

Implementation science: Studies examining adherence, provider adoption, healthcare-system integration, and cost-effective delivery will be essential for translating theoretical benefits into practice.

Longitudinal validation: Long-term prospective studies following individuals from middle age through late life with regular DunedinPACE assessments and comprehensive outcome tracking would provide more definitive evidence on predictive validity and intervention efficacy.

### 4.5 Ethical and practical considerations

The implementation of epigenetic-clock assessments and personalized aging interventions raises important ethical considerations. Privacy and consent frameworks must govern the collection, storage, and use of epigenetic data, and protections against discrimination based on biological-age assessments in insurance, employment, or healthcare-access contexts will be essential. Equity concerns also warrant attention, as access to advanced testing and intervention could exacerbate existing disparities if not thoughtfully distributed. From a practical perspective, barriers include limited provider awareness of epigenetic-aging concepts, insufficient technical infrastructure for routine assessment, and the absence of established clinical guidelines for interpreting and acting upon DunedinPACE results. Coordinated efforts across research, clinical, policy, and industry domains will be necessary to overcome these challenges.

## 5 Conclusion

We have developed a novel biomarker-based monitoring and intervention strategy for longevity medicine based on routine DunedinPACE assessments in clinical settings, establishing a quantitative framework linking epigenetic aging pace to frailty progression and economic outcomes. Our modeling indicates that intervention scenarios targeting DunedinPACE control yield savings relative to baseline in both healthcare costs and quality of life, more pronounced over time as intervention effects accumulate. The return on investment appears favorable from the individual perspective and, from the healthcare-system perspective, contingent on the cost of measurement, with specific values depending on implementation parameters and economic assumptions.

A key limitation of our current approach is its focus on caloric-restriction effects on the frailty pathway, which is one facet of a multidimensional relationship between aging interventions and age-related pathologies. Future research should expand this framework to encompass other intervention modalities and specific disease trajectories, particularly neurological, cardiovascular, and musculoskeletal conditions. Additionally, the cross-cohort extrapolation between Mak et al. (2023) and the CALERIE trial introduces uncertainty that should be addressed through further validation.

The primary contribution of this work is a translational bridge between molecular-level aging biomarkers and economic healthcare metrics via the frailty pathway, demonstrating how emerging aging-assessment technologies might facilitate earlier, more effective intervention. As population aging accelerates globally, evidence-based strategies to extend healthspan become increasingly urgent, and epigenetic pace-of-aging assessments may offer a valuable tool in this endeavor.

## Data Availability

Publicly available datasets were analyzed in this study. This data can be found here: https://github.com/felizzi/international-gains-to-healthy-longevity.
